# Transient Horner Syndrome Following Interscalene Nerve Block for Rotator Cuff Repair: A Case Report

**DOI:** 10.7759/cureus.115896

**Published:** 2026-09-07

**Authors:** Lalitha Battineni, Ruiqing Sun, Joseph Lin, Aabhishkar Bhattarai, Ahmad Shawagfeh

**Affiliations:** 1 Neurology, University of Texas Medical Branch (UTMB), Galveston, USA

**Keywords:** anhidrosis, hypothalamus, interscalene brachial plexus block, miosis, oculosympathetic paresis, partial ptosis, rotator cuff repair surgery, sympathetic innervation, transient horner syndrome, ultrasound-guided interscalene catheter

## Abstract

Horner syndrome is a well-recognized constellation of symptoms resulting from the disruption of the sympathetic pathway. It may occur in a variety of clinical contexts, including trauma, tumors, or iatrogenic causes. We report a rare case of transient Horner syndrome following rotator cuff repair with interscalene nerve block. Imaging ruled out structural or vascular causes. The syndrome resolved spontaneously by postoperative day 3. This case highlights the importance of clinician awareness regarding this benign but alarming postoperative complication.

This case was presented as a meeting abstract at the 2020 American Academy of Neurology (AAN) Annual Scientific Meeting, April 25 to May 1, 2020.

## Introduction

Horner syndrome is a neuro-ophthalmologic condition that results from the disruption of the sympathetic innervation to the eye and face, producing a classic triad of miosis, ptosis, and anhidrosis, although the presence of anhidrosis may vary based on anatomy and the location of injury. These signs reflect impaired sympathetic activity affecting the pupil dilator, Müller’s muscle of the upper eyelid, and facial sweat glands, respectively. Additional signs may include enophthalmos and the elevation of the lower eyelid (“reverse ptosis”) [1]. The common causes of Horner syndrome include lesions of the hypothalamus and brain stem, encephalitis, meningitis, multiple sclerosis, syrinx, spinal cord tumor, brachial plexus trauma, Pancoast tumor, the infection of the lung apex, carotid artery dissection or aneurysm, cavernous sinus lesions, cluster headaches or migraine, herpes zoster infection, and temporal arteritis [2].

While potentially alarming, Horner syndrome may arise from benign iatrogenic causes, particularly in the context of regional anesthesia. The interscalene brachial plexus block, commonly used for shoulder surgeries, has been associated with transient Horner syndrome due to anesthetic spread to the nearby sympathetic chain [3]. While the immediate, short-lived occurrence of Horner syndrome is well-characterized in the literature, atypical presentations marked by a substantial delay in onset and persistence beyond the pharmacological duration of the local anesthetic remain poorly documented. Such atypical timelines create a critical diagnostic dilemma: clinicians must distinguish benign, delayed fascial drug migration from serious procedure-related neurovascular emergencies, most notably iatrogenic internal carotid artery dissection. This report describes a case of delayed-onset, prolonged Horner syndrome following an ultrasound-guided interscalene block, highlighting the possible anatomical mechanisms of delayed fascial spread, the diagnostic framework for ruling out secondary vascular trauma, and adding to the growing literature supporting this late-onset benign Horner syndrome.

## Case presentation

A 60-year-old man with a medical history of dyslipidemia and benign prostatic hyperplasia underwent elective arthroscopic rotator cuff repair of the right shoulder. The patient was not receiving antiplatelet or anticoagulant therapy, and coagulation parameters were within normal limits. Preoperatively, he received an ultrasound-guided interscalene nerve block. A stimulation needle and catheter were placed in the interscalene groove formed by the anterior scalene muscle medially and the middle scalene muscle laterally, deep to the sternocleidomastoid muscle (SCM) and prevertebral fascia under ultrasound guidance. After a confirmed negative aspiration from the interscalene, 30 mL of 0.5% ropivacaine with epinephrine (1:200,000) was administered by continuous infusion. No paresthesia on initial incremental injection was noted. General anesthesia was then induced. The surgery was uneventful and lasted approximately 90 minutes. He was discharged the same day without complications.

Approximately seven hours postoperatively, the patient noticed right eye drooping and blurred vision. He denied double vision, tearing, eye pain, facial numbness, or facial anhidrosis. He reported a baseline history of occasional, mild, bilateral tension-type headaches that occurred preoperatively and remained unchanged in quality, frequency, and severity postoperatively. The patient had no history of recent cervical trauma, chiropractic manipulation, vigorous physical strain, or unusual intraoperative neck hyperextension or rotation.

The following day, the persistent symptoms led him to seek evaluation by his primary care physician, who observed right upper eyelid ptosis and miosis and referred him to ophthalmology. The ophthalmologist suspected Horner syndrome and referred the patient to the emergency department for further workup.

On physical examination, vital signs were stable, and the patient was neurologically intact except for the following: partial right-sided ptosis (~2 mm), the elevation of the right lower eyelid, and anisocoria with a right pupil of 2 mm and a left pupil of 3.5 mm in ambient light, more pronounced in dim lighting. There was equivocal dilation lag. Visual acuity and extraocular movements were intact bilaterally.

Given the concern for potentially life-threatening causes, a comprehensive imaging workup was performed. A CT angiography of the head and neck (Figure 1), an MRI of the brain (Figure 2), and a CT of the chest (Figure 3) revealed no evidence of intracranial pathology, carotid artery dissection, mediastinal masses, or apical lung mass.

**Figure 1:**
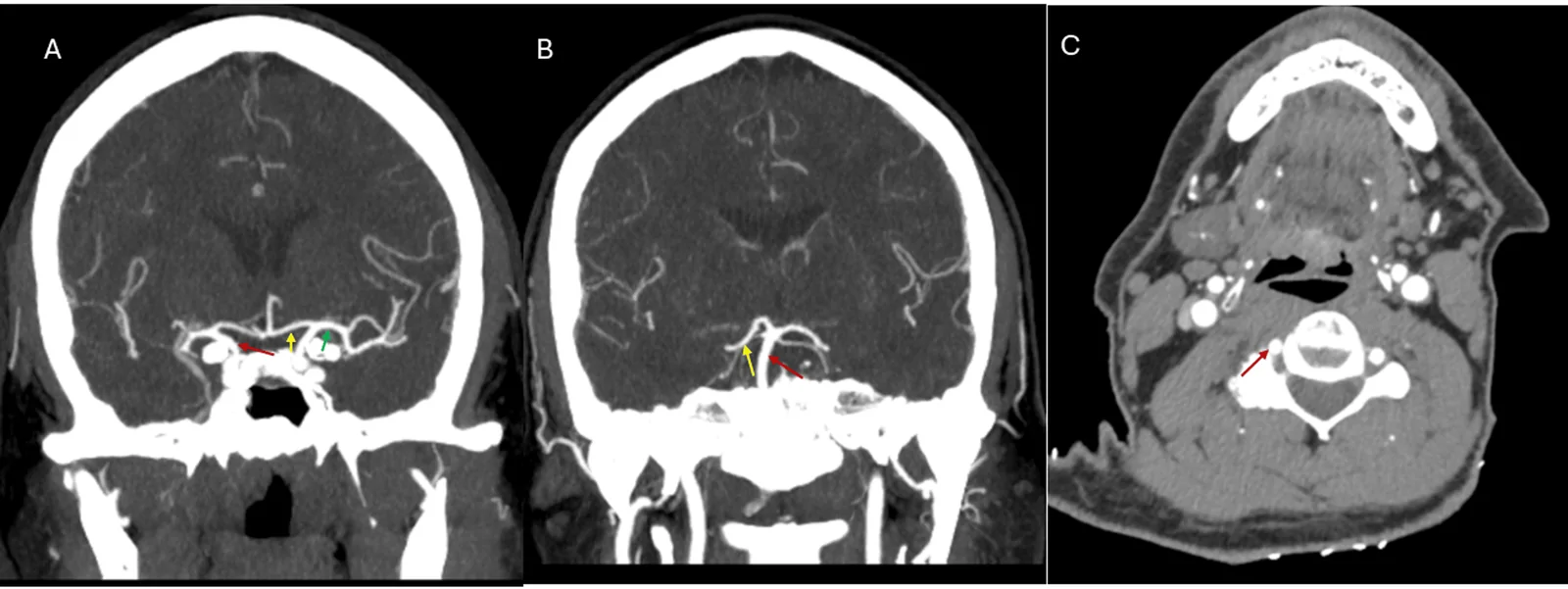
CT angiogram of the head (A and B) and neck (C) (A) Right internal carotid artery (red arrow), left anterior cerebral artery (yellow arrow), and left middle cerebral artery (green arrow); (B) basilar artery (red arrow) and right posterior cerebral artery (yellow arrow); (C) right internal carotid artery (red arrow). There is no high-grade stenosis, aneurysm, or vascular malformation

**Figure 2:**
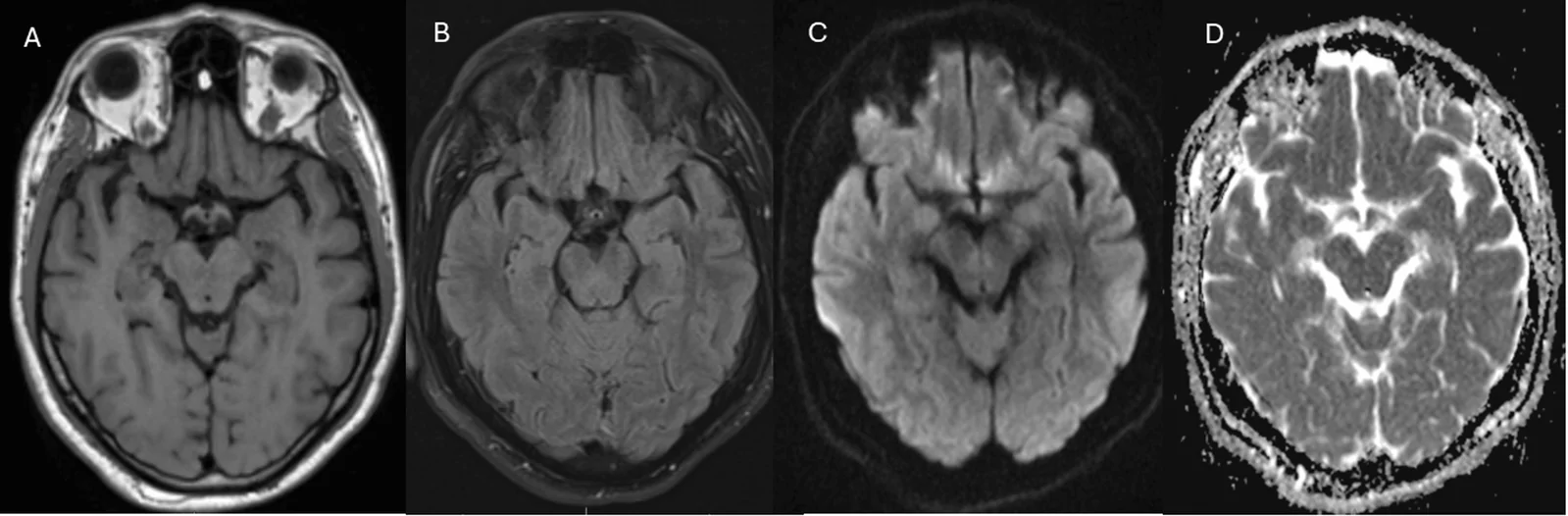
Normal brain MRI: T1 (A), FLAIR (B), DWI (C), and ADC (D) Normal gray-white matter differentiation throughout the cerebral hemispheres, cerebellum, and brainstem. No white matter changes. No evidence of acute infarction, acute intracranial hemorrhage, mass effect, or midline shift. No restricted diffusion on DWI/ADC (C/D) FLAIR, fluid-attenuated inversion recovery; DWI, diffusion-weighted imaging; ADC, apparent diffusion coefficient

**Figure 3:**
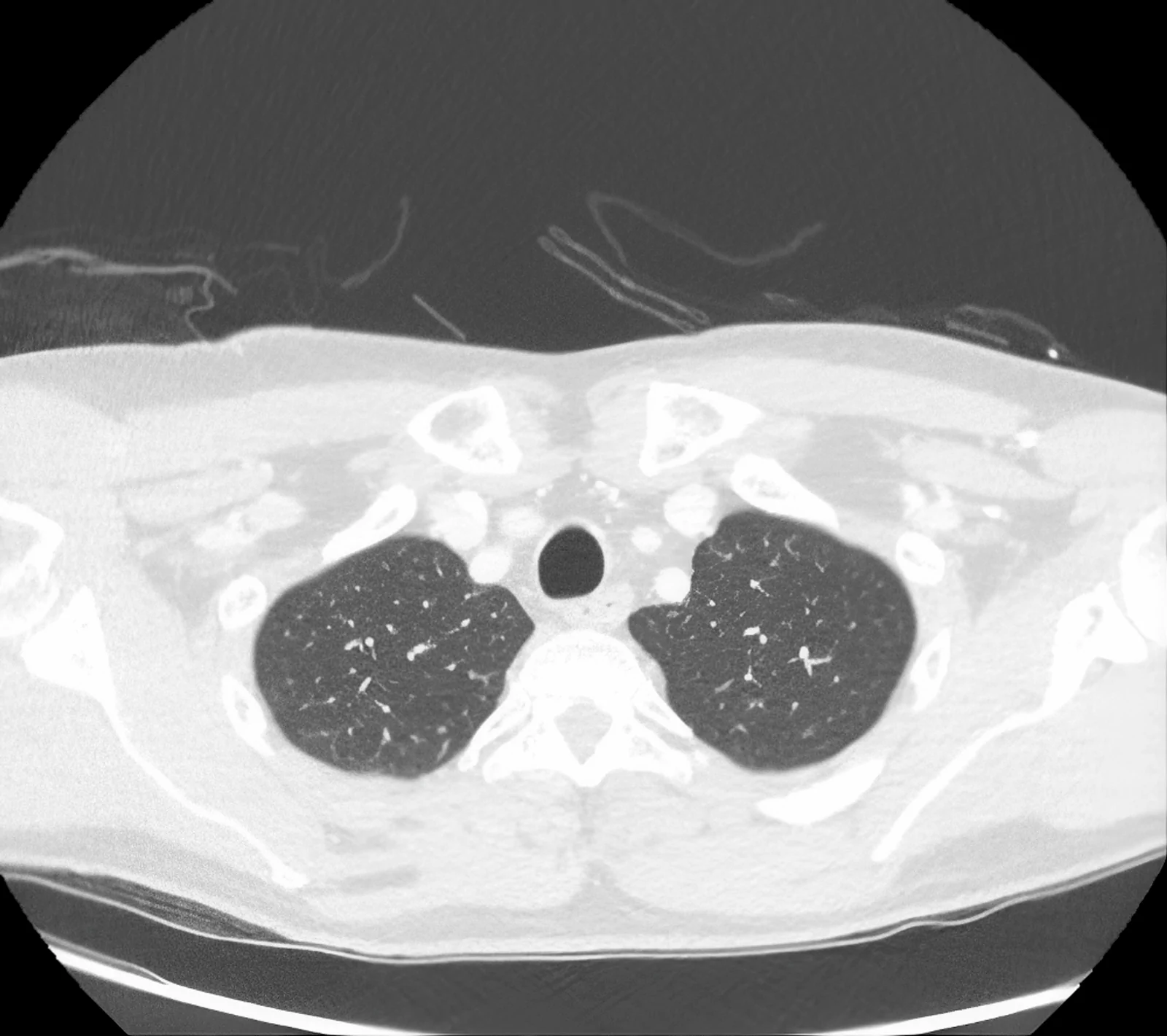
Normal chest CT with contrast Normal SVC and central mediastinal veins. No mediastinal masses or Pancoast tumors. No CT findings to explain the patient’s Horner syndrome SVC: superior vena cava

With structural, neoplastic, and vascular causes excluded and considering the temporal relationship to the regional block, the patient was diagnosed with transient Horner syndrome possibly due to local anesthetic spread to the cervical sympathetic chain. He was reassured and discharged home without intervention. The symptoms resolved completely by postoperative day 3.

## Discussion

Horner syndrome arises from lesions affecting any part of the oculosympathetic pathway, which consists of three distinct neurons extending from the hypothalamus to the orbit [4,5]. First-order (central) neurons originate in the hypothalamus and descend through the brainstem to the ciliospinal center of Budge in the spinal cord at levels C8-T2. Second-order (preganglionic) neurons exit the spinal cord at T1, ascend over the apex of the lung, and synapse in the superior cervical ganglion located near the carotid bifurcation. Third-order (postganglionic) neurons exit from the superior cervical ganglion and travel along the internal carotid artery into the cavernous sinus, joining the ophthalmic division of the trigeminal nerve to innervate the iris dilator muscle, Müller’s muscle, and sweat glands (Figure 4).

**Figure 4:**
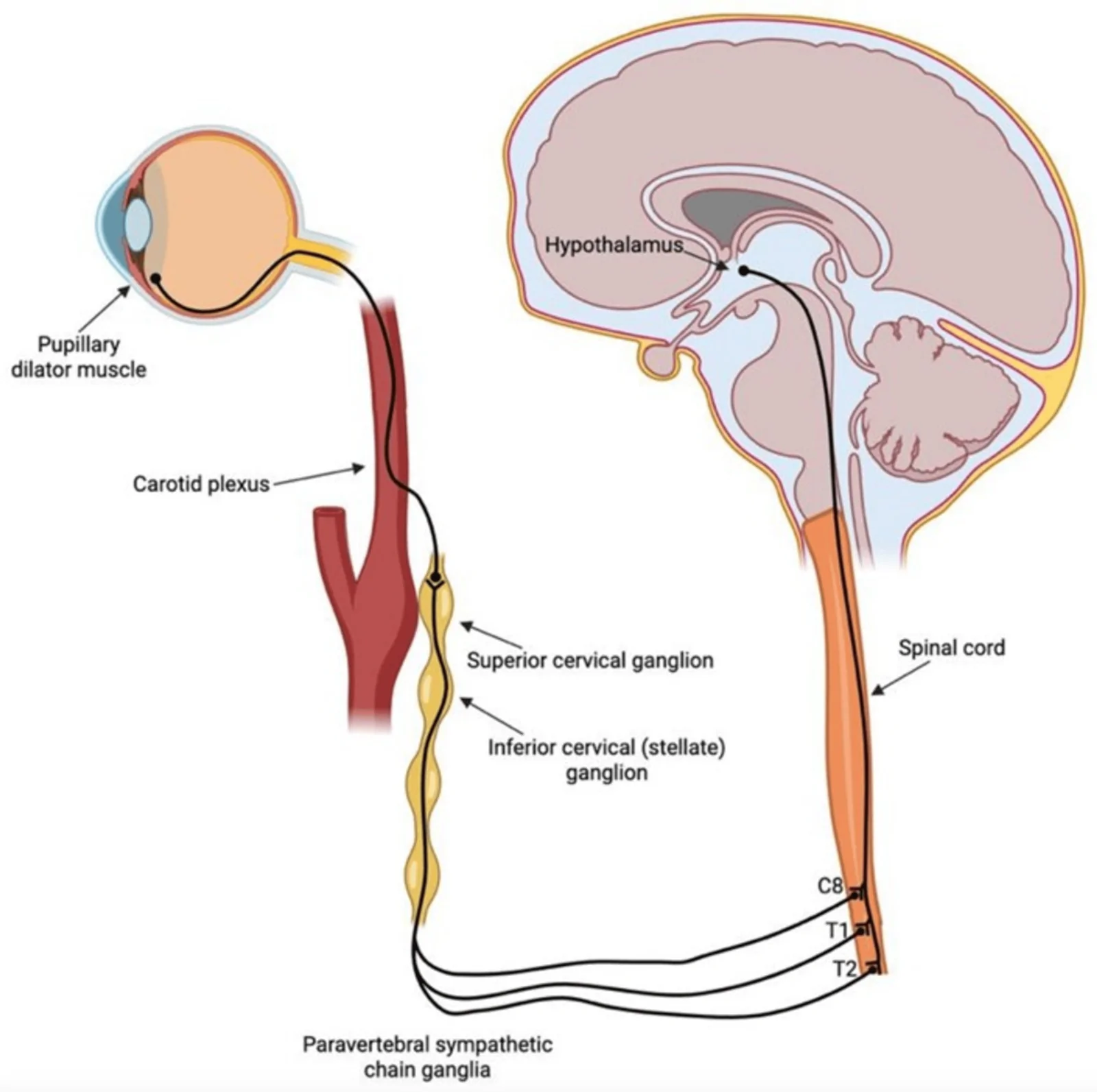
Sympathetic pathways causing pupillary dilation First-order neuron in the hypothalamus; second-order neuron at levels C8, T1,  and T2 in the spinal cord; third-order neuron in the superior cervical ganglion. Created in BioRender by Sun R (2026) (https://BioRender.com/1o1fyhw)

The clinical presentation may vary depending on the location of the lesion. Lesions involving first-order neurons (central) are often associated with additional neurologic deficits due to brainstem or spinal cord involvement. Second-order lesions typically occur in the thoracic outlet, often related to trauma or tumor. Third-order lesions, particularly those involving the internal carotid artery, may result from dissection, migraine, or cavernous sinus pathology [4,5].

Transient Horner syndrome is a well-documented but under-recognized complication of interscalene and supraclavicular brachial plexus blocks. Incidence rates vary depending on volume and concentration of the anesthetic agent and the technique of the brachial plexus blocks [6-9]. In contrast to the typical clinical trajectory, wherein Horner syndrome manifests within minutes of injection and resolves alongside the somatic nerve block within 12-24 hours, our patient exhibited a notably delayed onset of seven hours and an atypical duration spanning three days [3,6-8].

Given the unusual presentation of our case, it is essential to distinguish the benign postoperative complication from potentially life-threatening pathology, including tumors of the lung apex, carotid artery dissection, neck hematoma, and demyelinating diseases. As demonstrated by Padaki et al., mechanical interventions in the neck, including interscalene brachial plexus blocks, can rarely precipitate iatrogenic arterial dissection [10]. In addition, Ekatodramis et al. reported prolonged Horner syndrome due to neck hematoma after continuous interscalene block [11]. The patient’s brain MRI, CT angiography of the head and neck, and CT of the chest were normal, which made potential ominous etiology less likely. It is important to acknowledge that CT angiography is less sensitive than axial T1-weighted fat-suppressed MRI for detecting non-stenosing intramural hematomas.

While Horner syndrome typically subsides promptly with the resolution of anesthesia, long-lasting Horner syndrome is rarely reported in the literature [12,13]. Sukhani et al. [12] reported a case of Horner syndrome after an interscalene block had been present for at least one month, and McNeely et al. [13] demonstrated that Horner syndrome lasted more than two months. These two cases raised the possibility of traumatic or chemical injury to the sympathetic nerve fibers due to their long duration. The atypical course of our case might be explained by a slow, progressive fascial migration of long-acting ropivacaine along the prevertebral fascia toward the sympathetic trunk and stellate ganglion, rather than immediate needle-tip spread or direct mechanical trauma. Furthermore, anatomical compartmental sequestration within the deep cervical fascial planes may establish a localized drug depot, sustaining the blockade of unmyelinated sympathetic C-fibers (postganglionic sympathetic fibers) well after large myelinated somatic sensory-motor fibers have fully regained function. Higher concentration and total volume of ropivacaine used in our patient might promote wider fascial diffusion and prolong sympathetic blockade.

## Conclusions

This case underscores late-onset and prolonged Horner syndrome as a rare but benign complication of interscalene brachial plexus block. The recognition of this condition is vital for clinicians performing or managing patients undergoing regional anesthesia. However, clinicians must maintain vigilance when the clinical timeline is atypical: the confirmation of a benign etiology relies upon the exclusion of an ominous etiology. Once serious pathology is ruled out, reassurance and observation are generally sufficient. This case reinforces the importance of anatomical knowledge and the awareness of benign predictable complications of regional anesthesia.
